# Closed-loop artificial intelligence agents for animal epidemic prediction and decision support in livestock farming: a review

**DOI:** 10.3389/fvets.2026.1968632

**Published:** 2026-09-23

**Authors:** Yuzhi Wang, Yingtong Zhou, Liyu Li, Huiling Xu, Wangze Ni, Xiaoliang Li

**Affiliations:** 1 Department of Veterinary Medicine, College of Animal Sciences, Beijing Life Science and Technology Research Institute-Zhejiang University Joint R&D Center, Zhejiang University, Hangzhou, Zhejiang, China; 2 College of Biosystems Engineering and Food Science, Zhejiang University, Hangzhou, Zhejiang, China; 3 Department of Electrical and Computer Engineering, Faculty of Science and Technology, University of Macau, Taipa, Macao SAR, China; 4 College of Computer Science and Technology, Zhejiang University, Hangzhou, Zhejiang, China; 5 The Rural Development Academy, Zhejiang University, Hangzhou, Zhejiang, China

**Keywords:** AI agents, animal epidemic prediction, automated diagnostics, closed-loop surveillance, precision livestock farming

## Abstract

Animal diseases continuously threaten livestock production and public health, yet current surveillance approaches remain largely passive and fragmented. Conventional artificial intelligence models typically function as open-loop predictors, delivering static outputs that fail to accommodate the dynamic operational needs of veterinarians in real-world farm settings. This review explores an emerging paradigm of AI agents for animal disease monitoring, emphasizing how such agents can transition from passive observation to active intervention through a closed-loop perception–decision–feedback architecture. We first classify multi-scale sensing technologies: at the micro-scale, automated molecular diagnostics and biosensors; at the macro-scale, computer-vision-based phenotyping. These technologies collectively provide the data streams required for continuous surveillance. The review then discusses how multi-modal inputs, time-series forecasting, resource-aware decision-making, and reinforcement learning can be integrated into an agent-based workflow. Importantly, most current veterinary research evidence supports only the individual components of this framework, rather than the deployment of fully autonomous, iteratively evolving closed-loop agents in actual field environments. Translating closed-loop AI from a conceptual framework into a reliable veterinary decision-support tool will therefore require further advances in validation, standardization, governance, and human oversight.

## 1 Introduction

In recent years, outbreaks of African swine fever (ASF), highly pathogenic avian influenza (HPAI), foot-and-mouth disease (FMD), and porcine reproductive and respiratory syndrome (PRRS) have shown that animal epidemics remain a major challenge to livestock production, animal welfare, food security, and public health. These diseases spread rapidly, are often associated with high mortality, and frequently cause trade disruption and production losses. Between 2021 and 2023, ASF was reported in 50 countries and resulted in the death of more than 1.5 million pigs worldwide (1). Likewise, from 2005 to 2024, more than 633 million poultry died or were culled globally because of HPAI. In intensive production systems, high stocking density and extended supply chains make early detection increasingly difficult (2, 3). Under these conditions, animal health diagnostics are no longer expected merely to confirm the presence of disease. There is growing demand for surveillance approaches that can detect abnormal patterns at an earlier stage, support near real-time risk assessment, and provide a basis for timely intervention (4, 5).

Traditional diagnostic methods for animal epidemics can generally be classified into molecular diagnostics, immunological diagnostics, and pathogen-based diagnostics according to their underlying principles. These methods remain indispensable, but their limitations are also evident. In clinical practice, disease recognition still relies mainly on clinical observation and laboratory diagnosis (4). In many situations, such approaches are reactive rather than proactive, and they are often too slow when infection remains subclinical or when early signs are mild or atypical. In ASF, for example, by the time obvious mortality patterns or hemorrhagic signs are recognized, infection may already have spread within or between farms. In brucellosis, serological tests may yield false-negative results in the early stage of infection because antibody levels have not yet reached detectable levels (6). In addition, diagnostic capacity may be restricted by infrastructure, transport, and shortages of trained personnel, especially when surveillance must cover large populations under heterogeneous farm conditions. Recent reviews have also shown that many surveillance systems remain fragmented, weakly integrated, and difficult to evaluate consistently, which reduces their value for early warning and risk assessment (7–9).

Meanwhile, routine diagnostics, herd records, sensor outputs, images, behavioral observations, and environmental measurements are increasingly being generated as structured or semi-structured data streams. Research in precision livestock farming has shown that these sources can improve the continuous monitoring of animal health and welfare and may detect changes earlier than routine inspection alone (10). Nowadays, artificial intelligence -based methods are becoming increasingly relevant in animal production settings. However, data abundance alone does not improve epidemic management. The key challenge is whether these heterogeneous sources of information can be interpreted in a biologically meaningful and operationally useful way under real veterinary conditions (2, 11).

Against this backdrop, artificial intelligence agents (AI agents) are increasingly drawing attention. Conventional AI methods (e.g., deep neural networks, large language models) can already integrate heterogeneous data for prediction, so the rationale for an agent-based approach is not data fusion alone. In this review, we use the term veterinary AI agent to describe a closed-loop decision system that can perceive multi-scale data, reason about epidemic trajectories, propose resource-aware actions (e.g., targeted sampling or isolation), and update subsequent decisions from observed outcomes. In current veterinary practice, however, most of these capabilities have been demonstrated as separate components rather than as fully integrated autonomous systems.

The specific objective of this narrative review is therefore to examine which technical components are already supported by veterinary or animal-health evidence, how they could be integrated into a closed-loop decision framework, and where empirical field validation is still lacking. This review mainly addresses one question: How can multi-scale diagnostic and phenotypic data be integrated with dynamic feedback to support veterinary disease surveillance? To address this question, Section 2 reviews micro-level biochemical sensing, Section 3 reviews macro-level phenotyping, and Section 4 presents a prospective closed-loop agent framework that links these inputs to sequential decision support under real-world resource constraints.

To make this narrative review more transparent, we mainly searched for relevant literature published between January 2015 and June 2026 on PubMed, Web of Science, and Scopus. The search keywords included artificial intelligence, machine learning, deep learning, reinforcement learning, AI agents, veterinary, animal diseases, animal husbandry, epidemiological surveillance, diagnosis, and precision livestock farming. We prioritized studies related to AI-based animal health monitoring, diagnosis, prediction, or decision support, and also checked the reference lists of key reviews to find more publications.

## 2 AI diagnosis and monitoring based on automated detection data streams

To better understand animal infectious diseases, we first examine small-scale biological changes at the molecular and biochemical level. Laboratory testing and field-based rapid testing have long served as the cornerstone of animal epidemic surveillance systems. However, their role is rapidly expanding from providing intermittent confirmation to continuously generating structured data that can support higher-level risk prediction systems. Automated detection technologies encompass immunological methods such as ELISA and lateral flow assays, nucleic acid amplification techniques (PCR, ddPCR, and isothermal amplification), biosensors, and CRISPR diagnostic platforms. These approaches can generate high-frequency signals reflecting pathogen presence, host immune responses, and increasingly host susceptibility markers. When these outputs are collected and aggregated together with standardized metadata (time, location, animal ID, sample type, reagent/platform information, etc.) they can be regarded as biochemical indicator data streams suitable for machine learning integration and modeling, thereby providing an input foundation for intelligent-agent-enabled epidemic risk prediction.

### 2.1 From diagnostic results to data streams

Molecular diagnostics have long been recognized for their ability to identify pathogens before the full manifestation of clinical symptoms, thereby opening earlier windows for intervention. This principle has been systematically discussed in foundational reviews of molecular veterinary diagnostics (12, 13). The current diagnostic landscape is characterized by the parallel development of expanded central laboratory capacity and decentralized detection. On one hand, confirmatory capabilities such as sequencing are being further strengthened and expanded. On the other hand, molecular detection is increasingly moving toward field settings. For example, the integration of conventional (RT-) PCR products with portable nanopore sequencing enables rapid identification of ASFV, CSFV, and FMDV under resource-limited conditions while also providing phylogenetic interpretation. This approach demonstrates how confirmatory workflows can be transformed into operational near-real-time molecular monitoring signals (14). Such strategies are particularly important for transboundary animal epidemics because rapid identification directly determines the speed of control responses.

At the same time, immunological rapid detection technologies are evolving toward greater speed, portability, and multiplexing capability, enabling actionable antigen or antibody signals to be generated even in environments lacking full laboratory infrastructure. Fluorescent microsphere immunochromatographic assays (FM-ICA) for SADS-CoV have shown high concordance with qPCR while providing shorter detection times and simpler operation, indicating that immunological detection can support early screening and monitoring during epidemic emergence (15). Quantum-dot dual-channel immunochromatographic sensors developed for ASF and pseudorabies can produce results in approximately 20 min and demonstrate good agreement with commercial diagnostic kits, making them suitable for continuous population-level monitoring (16). In addition, LFIA/LFA technologies are increasingly applied in the detection of zoonotic and animal viruses. Although their sensitivity and detection spectrum still require improvement, their speed and deployability have made them an important form of field detection (17). Immunological rapid tests designed for multi-host applications are also emerging. For instance, chimeric protein A/G lateral flow assays exploit cross-species IgG binding properties to enable screening for brucellosis in livestock, providing a practical tool for multi-species monitoring (18).

Biosensors represent another frontier for the generation of diagnostic data streams. Reviews of biosensor technologies in livestock and poultry indicate that electrochemical sensors, quantum-dot fluorescence systems, and carbon nanotube field-effect transistor (FET) platforms can use antibodies, nucleic acids, or aptamers as recognition elements to detect pathogen antigens, antibodies, or nucleic acids. These approaches often provide faster detection than traditional methods and exhibit strong analytical performance, although they remain constrained by background interference, sample stability in complex matrices, and scalability of production (19, 20). CRISPR diagnostics further integrate sequence-specific recognition with portable readout technologies. Electrochemical CRISPR sensors have demonstrated potential for rapid detection and have been proposed for field and wildlife monitoring. However, their effective deployment depends on standardized protocols and improved resistance to environmental interference (21, 22).

### 2.2 Host–pathogen integration and prediction-relevant biomarkers

A key step in moving from diagnosis to prediction is the integration of pathogen signals with host biomarkers that determine susceptibility, progression rate, and transmission potential. A representative example is the quadruplex droplet digital PCR (ddPCR) assay developed for bovine leukemia virus (BLV). This method simultaneously quantifies pathogen load (proviral load) and genotypes host susceptibility or resistance alleles (DRB3 variants) within a single reaction and shows high agreement with ELISA and qPCR results (23).

Such combined detection outputs produce multidimensional biomarkers that can be used for risk stratification at both individual and population levels, naturally aligning with the state variables required for predictive and decision-oriented systems. This trend is consistent with the broader consensus in biomarker research: when clinical symptoms appear late, present atypically, or vary substantially across breeds, molecular biomarkers become particularly important for early identification. High-throughput omics platforms (genomics, proteomics, and metabolomics) are increasingly used to discover disease-specific molecular signatures, while emerging technologies (microfluidics, aptamer-based sensing, and AI-enhanced imaging) may further advance early detection and mechanism-oriented diagnostics (24). However, methodological reviews in metabolomics emphasize that reliable data streams must be built upon standardized extraction protocols, rigorous quality control, normalization procedures, and inter-laboratory reproducibility. Without such methodological rigor, machine learning models may inadvertently learn technical variability rather than epidemic biology (25).

Non-invasive sampling strategies further improve the feasibility of continuous monitoring. Saliva has been proposed as an animal-friendly biological fluid containing biomarkers associated with infection, immunity, stress, and inflammation. Because saliva sampling is inexpensive, minimally stressful for animals, and easy to perform by non-specialists, it can support large-scale repeated sampling and is therefore suitable for early screening and health monitoring during epidemic emergence (26). From a systems engineering perspective, such sampling approaches reduce the friction cost of longitudinal monitoring and represent an important enabling condition for early warning.

### 2.3 Integration of high-dimensional diagnostic data streams and early-warning challenges

The value of machine learning in automated detection data streams is mainly reflected in three aspects:

(1) data integration across platforms and indicators;
(2) anomaly pattern detection and early risk scoring;
(3) operational decision support for field deployment.

Evidence from production environments suggests that automated detection systems can change the timing of intervention and improve outcomes. A feedlot study comparing automated animal epidemic detection (ADD) with traditional pen-riding monitoring showed that ADD reduced the time to first treatment for bovine respiratory epidemic (BRD), decreased mortality and removal rates, and increased economic returns. These findings suggest that sensor-based continuous monitoring can significantly enhance practical surveillance performance (27). Although the study did not isolate the contribution of specific machine learning architectures, the results support the broader conclusion that stream-based monitoring can improve epidemic management performance.

Nevertheless, significant barriers remain before scalable real-time early warning can be achieved. First, field conditions may introduce performance drift. Although biosensors provide high sensitivity and rapid detection, they can be susceptible to environmental interference and stability limitations in complex settings (19). Second, many POCT technologies face a gap between laboratory validation and field performance. Reviews focusing on low- and middle-income countries indicate that numerous veterinary POCT devices lack evaluation in real-world conditions outside laboratory environments, and the absence of mandatory validation regulations allows substandard products to enter the market, potentially weakening epidemic control efforts (28). Third, standardized evaluation must extend beyond simple measures of accuracy. The FIT-REASSURED framework emphasizes that the evaluation of veterinary POCT should consider not only sensitivity and specificity but also usability, robustness, cost, ease of operation, and real-time connectivity, in order to meet the requirements of digital surveillance systems for reliable input data (29). These deployment constraints directly determine whether diagnostic data streams can provide stable and reliable inputs for higher-level predictive AI agents.

In addition, surveillance during epidemic outbreaks is strongly constrained by resource capacity. Simulation studies of ASF spread in high-density swine-production regions indicate that the number of sampling personnel and laboratory testing throughput may become critical bottlenecks, potentially causing large-scale delays. At the same time, modifying sampling strategies such as using oral fluid can significantly reduce labor and resource requirements (30). These findings emphasize that early warning is not purely an algorithmic problem but rather a socio-technical coupled system whose timeliness depends on logistics, personnel, and laboratory capacity. Figure 1 shows the transformation process from microdata collection to the construction of predictive factors.

**Figure 1:**
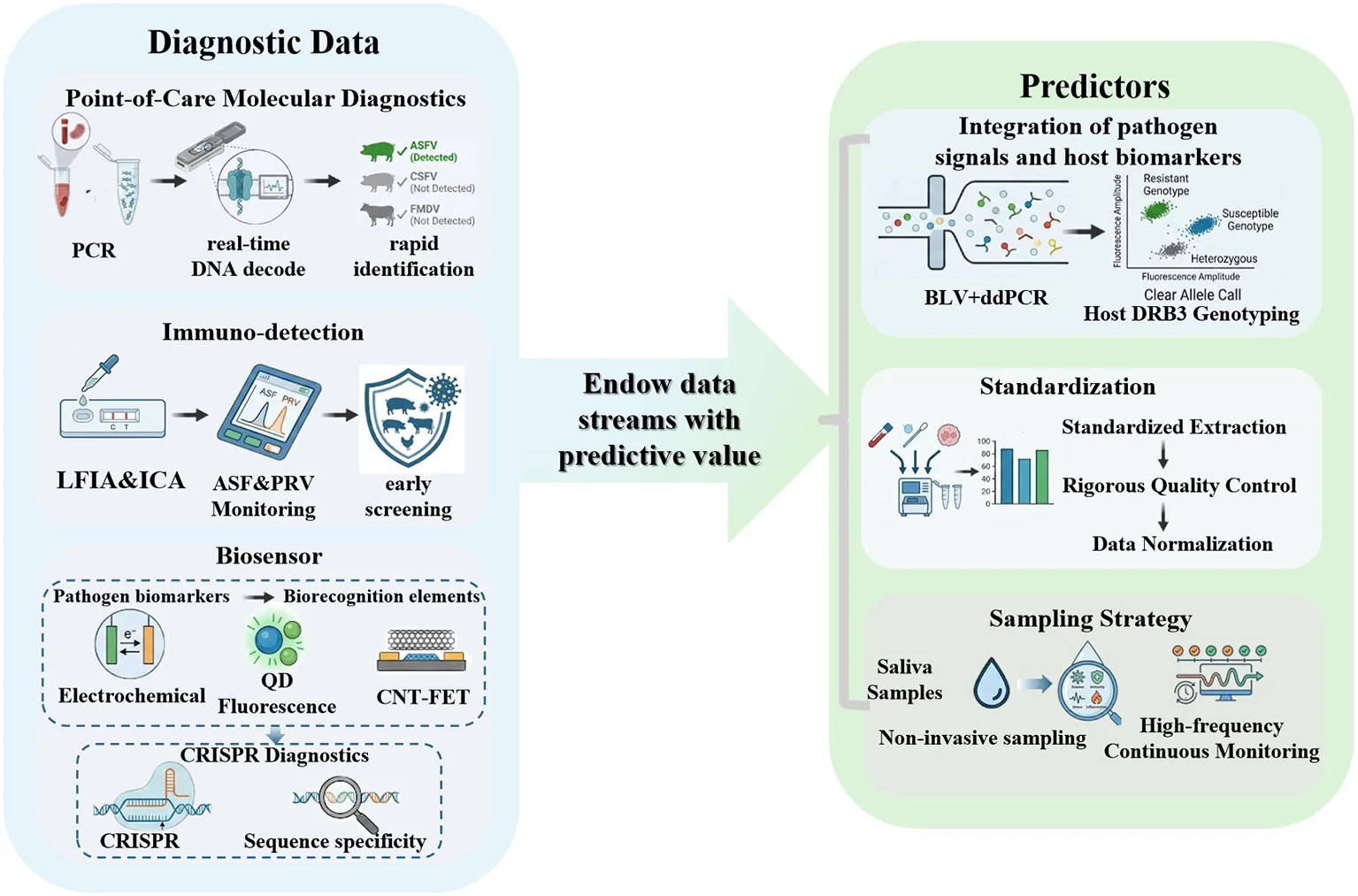
Framework for transforming multi-source diagnostic data into reliable predictive factors. **(Left)** Diagnostic data: Multiple point-of-care (POC) technologies are included, covering molecular diagnostic techniques (polymerase chain reaction, PCR; real-time decoding technologies), immunological detection methods (lateral flow immunoassays, LFIA; immunochromatographic assays, ICA), and advanced biosensors (electrochemical sensors and clustered regularly interspaced short palindromic repeats, CRISPR). These technologies enable rapid identification of pathogens such as African swine fever virus (ASFV) and foot-and-mouth disease virus (FMDV). **(Right)** Predictive factors: The central objective is to enhance data utility through three key strategies: (1) integrating pathogen load measurements with host genetic markers (e.g., *DRB3* genotyping); (2) establishing standardized protocols for extraction and data normalization to ensure data quality; (3) optimizing sampling strategies, prioritizing non-invasive, high-frequency monitoring approaches (e.g., saliva sampling) to capture temporal dynamics.

## 3 Multimodal analysis of imaging and clinical data

Beyond these internal molecular changes, we then turn to visible signs and behaviors at the whole-animal level. The development of computer vision (CV) and deep learning (DL) has provided, for the first time, a technological basis for the visualization, quantification, and continuous monitoring of clinical manifestations of animal epidemics. By systematically modeling appearance, behavior, physiological manifestations, and their temporal evolution, AI-driven visual technologies are transforming traditional experience-based subjective observation into a standardized data-stream analysis process.

### 3.1 Foundations of visual perception and three-dimensional morphological quantification

#### 3.1.1 Construction of deep learning–based visual perception capability

Convolutional neural networks (CNNs) have evolved into comprehensive perception systems capable of object detection, individual tracking, semantic segmentation, and pose estimation.

The You Only Look Once (YOLO) algorithm family is widely used for identifying and counting individuals in crowded environments (31). Combined with tracking algorithms like DeepSORT, these systems reconstruct animal movement trajectories to compute activity indices (32). Architectures such as U-Net and Mask R-CNN enable pixel-level separation of animals from complex backgrounds, providing a prerequisite for body measurement, body condition scoring (BCS), and lesion-specific analysis.

Vision Transformer (ViT) and Swin Transformer have markedly improved the robustness of animal recognition systems under complex lighting conditions and rapid movement. In high-density and strongly occluded breeding environments, Swin Transformer improves individual recognition accuracy by over 6% compared to traditional CNNs (33). Detection Transformer (DETR)-based models further improve detection consistency for overlapping individuals in complex backgrounds (34). To further alleviate occlusion and posture changes, multi-view fusion strategies have reduced individual missed detection rates from 11.3% to 3.8% in intensive pig farms (35).

#### 3.1.2 Body condition scoring and three-dimensional quantitative assessment

Body condition scoring (BCS) is a key indicator reflecting energy reserves and chronic health problems in animals. A rapid decline in BCS is often an early signal of metabolic disorders, such as ketosis, or chronic infections (36). Three-dimensional depth cameras installed above passageways can capture three-dimensional point cloud data of the animal's back. Algorithms analyze geometric features including spinal curvature and the prominence of hip bones to automatically calculate BCS values.

Structured-light systems and LiDAR are increasingly used for three-dimensional morphological reconstruction in animals. LiDAR can generate high-density point clouds (>100,000 points/s), enabling automated body condition analysis (37). Researchers have further integrated 3D point clouds with Transformer-based point-cloud models, increasing the consistency between automated BCS estimation and expert scoring to above 0.78 (38). Recent trends also include the introduction of continuous monitoring concepts, such as analyzing the dynamic changes of three-dimensional features within the lactation cycle of dairy cows through LSTM or Temporal ConvNet models, which can predict the risk of ketosis 5 to 7 days in advance (39). Emerging three-dimensional reconstruction approaches such as neural radiance fields (NeRF) have also been explored for modeling the surface structure of animals (40).

### 3.2 Multidimensional clinical symptom screening: from lesion imaging to thermography

#### 3.2.1 Recognition of visible lesions and pathological images

AI-based image analysis operates across multiple clinical modalities. In histopathology, CNNs automatically identify and quantify lung lesions in tuberculosis-infected mice (41, 42), while commercial AI software demonstrates high sensitivity in detecting cardiogenic pulmonary edema on canine thoracic radiographs (43).

For dermatological lesions and morphological analysis, many highly contagious diseases produce visible marks on the skin. In the case of lumpy skin disease (LSD) in cattle, CNN-based models can identify early skin nodules from standard RGB images with sensitivities approaching 95% (44). For dermatological diseases, emerging super-resolution reconstruction techniques can reconstruct finer skin textures from low-resolution surveillance video, improving the detectability of small lesions. In monitoring LSD, for example, ESRGAN-based super-resolution algorithms have been used to enhance the detection of micro-lesions smaller than 3 mm (45). In addition, hyperspectral imaging (HSI) of the face and limbs in livestock is increasingly used to identify skin inflammation, necrosis, and microvascular abnormalities, allowing AI systems to detect early signals of local tissue damage before lesions become visible to the naked eye (46).

#### 3.2.2 Fever and inflammation screening driven by infrared thermography

Fever is the most common systemic response to infection. Infrared thermography (IRT) enables non-contact body temperature monitoring by detecting radiated surface temperature. Recent AI systems increasingly adopt RGB–thermal fusion strategies. RGB images are first used to localize key facial regions—such as the medial canthus and the base of the ear, which serve as thermal windows—and thermal imaging data from these regions are then extracted, improving measurement stability and repeatability (47). Beyond absolute temperature, AI systems can also analyze thermal distribution patterns. For example, lameness-related inflammation often produces characteristic hotspots in the hooves, whereas mastitis leads to asymmetric temperature distributions in the udder. For early screening, the combination of multiscale CNN architectures and attention mechanisms further enhances sensitivity to local inflammatory hotspots. In mastitis detection, a Swin Transformer-based thermal map model can automatically learn udder symmetry features, increasing the detection accuracy of subclinical mastitis without visible swelling to approximately 92% (48).

Deep learning-based thermal–environment calibration networks improve IRT accuracy under practical farming conditions. Furthermore, jointly modeling IRT and respiratory frequency via spatiotemporal analysis detects respiratory abnormalities earlier (49).

### 3.3 Behavioral repertoires, social structures, and welfare monitoring

#### 3.3.1 Individual abnormalities and group social dynamics

Sickness behaviors, including lethargy, reduced appetite, and social withdrawal, often precede physiological symptoms. ResNet-based models enable real-time recognition of behaviors like lying, standing, feeding, and aggression in cattle (32). Additionally, DeepLabCut-based keypoint tracking reconstructs gait sequences and limb angles to detect lameness (50).

Time-series modeling of group behavior is increasingly recognized as an important direction for early epidemic prediction. Transformer-based temporal behavior encoding models can learn changes in behavioral rhythms from continuous monitoring data and have been used to identify mastitis risk in advance (51). In social animals, visually derived contact frequencies and spatial distances between individuals allow groups to be represented as dynamic graph structures. Graph neural networks (GNN) have been applied to identify individuals with potentially high transmission risk and abnormal social interaction patterns, providing support for modeling population-level epidemic dynamics (52). For example, in swine influenza studies, individuals showing markedly reduced social contact often exhibit behavioral abnormalities before clinical symptoms appear.

#### 3.3.2 Visual quantification of pain and stress states

AI-digitized grimace scales and facial-feature scoring systems identify disease states in cattle with foot-and-mouth disease. Landmark-based high-density facial capture detects subtle micro-expressions, such as orbital tightening and lip corner contraction, for precise pain recognition in sheep and horses (53).

For stress monitoring, real-time infrared tracking of pupil dilation and eye aperture changes provides continuous evaluation. Furthermore, multimodal Automatic Pain Recognition (APR) systems objectively assess pain intensity by fusing facial expressions, body language, physiological signals, and vocalizations; notably, combining acoustic signals with facial features modestly improves the F1-score of pain classification models (54).

### 3.4 Multimodal fusion and clinical decision support

#### 3.4.1 Multimodal fusion architectures

Single visual modalities risk false positives; therefore, integrating RGB, depth, thermal, and audio signals improves diagnostic specificity. While early and late fusion are common, Multimodal Transformers are increasingly applied to adaptively adjust modality weights based on changing environments. For instance, during pig respiratory monitoring, the model automatically shifts weight to visual inputs if environmental noise degrades audio quality (55).

To address inconsistencies in sampling frequencies among different sensors, temporal alignment networks have been proposed to synchronize multimodal signals (56). In veterinary scenarios where data resources are limited, multimodal knowledge distillation has been used to improve the deployment efficiency of lightweight models, enabling models running on mobile devices or farm cameras to retain 85–90% of the original performance despite significant parameter compression (57).

#### 3.4.2 Clinical decision support and limitations

In clinical diagnostic support, AI-based image recognition has achieved high performance in specific tasks. However, the current consensus is that AI should serve as a decision support tool rather than a replacement for veterinary expertise (58). The technology shows considerable potential for rapid screening in field settings. Lightweight models can be deployed on resource-limited mobile devices, enabling real-time image-based diagnosis. In practical applications, explainable AI (XAI) techniques—such as Grad-CAM—have become increasingly important because they highlight lesion regions that influence model predictions, thereby improving trust in AI systems (59). IoT-integrated multimodal AI systems now process real-time video, audio, temperature, and location streams to simultaneously monitor diarrhea, respiratory epidemics, and lameness in large-scale pig farms (60). Nevertheless, most existing visual models remain optimized for single-task objectives, with limited cross-scenario generalization and weak integration with mechanistic understanding. These limitations may restrict their broader system-level application in epidemic prediction.

Taken together, these biochemical and phenotypic data streams provide complementary perspectives on animal diseases, ranging from early molecular changes to whole-animal clinical manifestations. Figure 2 summarizes the classification of various macroscopic phenotypic techniques. To clarify how these multi-scale signals can be linked to the next stage of epidemic prediction and intervention support, Table 1 summarizes the main data sources and their functions.

**Figure 2:**
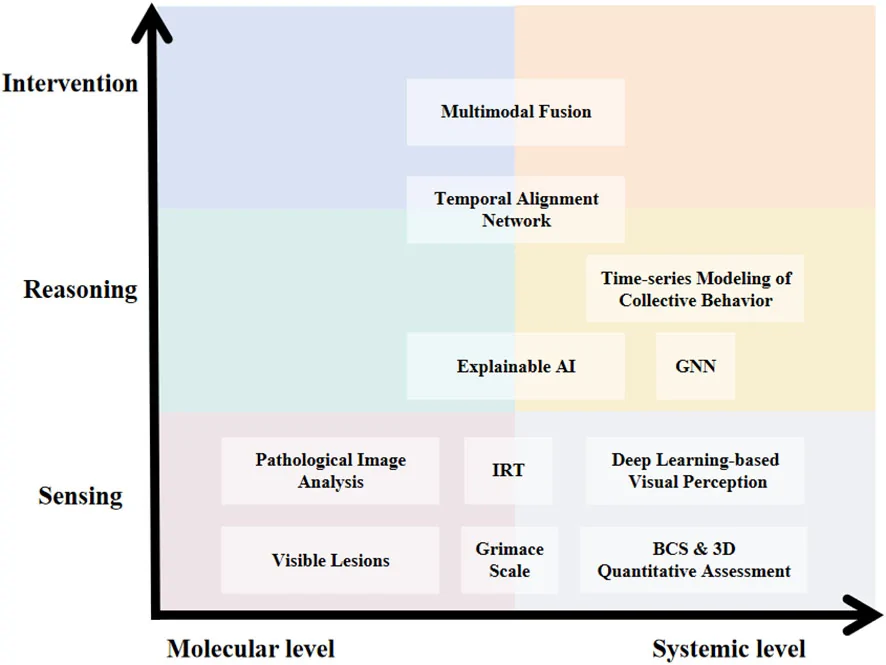
Technological taxonomy of intelligent-agent systems: a methodological mapping across functional and biological scales. This matrix classifies the core technologies along two dimensions. Vertical axis (functional evolution): from basic perception (data acquisition), progressing to inference (pattern analysis), and ultimately to intervention (decision-making). Horizontal axis (biological scale): covering the full spectrum from the molecular level (microscale pathology) to the system level (macroscale behavior and physiology). The figure illustrates how bottom-up perception technologies (e.g., pathological image analysis, deep learning–based visual perception) provide the foundation for higher-level inference models (e.g., graph neural networks, GNN; explainable artificial intelligence, XAI). These components are ultimately integrated through multimodal fusion, providing structured support for the formulation of intervention strategies.

**Table 1:** Multiscale data sources and their roles in animal epidemic surveillance, forecasting, and intervention support.

Scale	Data source	Representative technologies	Main information provided	Role in epidemic surveillance	Major limitations
Micro-scale	Automated diagnostic and biochemical data streams	PCR, qPCR, ddPCR, immunoassays, biosensors, CRISPR-based diagnostics, omics platforms	Pathogen presence, pathogen load, host immune responses, susceptibility-related biomarkers	Provides early biochemical evidence of infection and creates structured data streams for risk stratification before overt clinical signs appear	Data heterogeneity, limited standardization, quality-control requirements, and poor cross-platform comparability
Macro-scale	Clinical, imaging, and behavioral phenotypic data streams	Computer vision, CNNs, Transformer-based vision models, infrared thermography, depth imaging, pose estimation, multimodal sensing	Lesions, inflammation, fever, body condition, posture, activity, pain/stress, and other whole-animal phenotypes	Captures non-invasive phenotypic manifestations of disease progression at the whole-animal level and complements laboratory testing	Small veterinary datasets, interspecies and interbreed variability, insufficient external validation, and limited mechanistic interpretability
System level	Integrated surveillance data streams	Data integration frameworks, multimodal analysis, time-series prediction models, self-supervised learning, and decision-support systems	Epidemic trends, short- and medium-term risk prediction, intervention prioritization, and resource-aware recommendations	Integrates data from multiple sources to support continuous surveillance, risk forecasting, and intervention planning	Limited field validation, inconsistent evaluation standards, interpretability concerns, and deployment constraints related to regulation, logistics, and governance

## 4 Intelligent agent systems in epidemic prediction

The micro-biochemical detection and macro-phenotypic monitoring described in Parts 2 and 3 are more portable and scalable compared to traditional methods. However, in actual veterinary prevention and control scenarios, they still have limitations: Microscopic detection is mostly single-point discrete sampling, with insufficient stability and standardization, and can only provide molecular-level information, lacking linkage with animal conditions, environment and other variables; macro-phenotypic monitoring is easily interfered by interspecies differences and has insufficient phenotypic specificity. More importantly, both types of methods have the problem of data silos, unable to form a complete disease assessment chain, and lack dynamic decision-making and intervention priority planning under resource constraints, making it difficult to support timely and coordinated epidemic response. Recently, the rapid development of AI agents such as openclaw and Biomni has shown that AI agents have the powerful ability to process multi-source data and integrate microscopic and macroscopic data, which can effectively solve the problem of data silos in veterinary diagnosis. The integration and output of data by AI agents has become a promising solution. Accordingly, this section presents a prospective architecture assembled from component technologies with different levels of veterinary validation, rather than implying that end-to-end self-evolving veterinary agents are already routinely deployed.

### 4.1 From prediction to action: the paradigm shift enabled by AI agents

In this review, artificial intelligence agents in the veterinary field are redefined as closed-loop decision-making systems that not only integrate multi-source diagnostic and phenotypic data but also autonomously generate continuous intervention measures, evaluate their outcomes, and adjust strategies over time. Unlike traditional machine learning or deep learning models (such as long short-term memory networks, convolutional neural networks, and Transformers), which output static risk scores and then leave it to human experts to decide the next steps, these agents aim to answer: “Given the current uncertainties and limited resources, what specific actions should be taken next, and how should the strategy be adjusted upon observing the results?”

To achieve this, an AI agent in our animal epidemic scenario must perform a continuous four-step workflow:

Perception—Continuously absorb micro-scale (molecular diagnostics, biosensors) and macro-scale (computer vision, behavioral) data streams, converting them into a structured state representation (e.g., estimated infection prevalence, remaining testing capacity).

Reasoning—Use time-series foundation models and/or reinforcement learning to forecast near-future epidemic trajectories under different hypothetical actions.

Action—Under real-world constraints (sampling kits, laboratory throughput, personnel, logistics), output concrete, implementable intervention commands (e.g., “Prioritize oropharyngeal swabs from pen B-12,” “Request mobile lab for tomorrow,” “Initiate partial isolation of barn 3”).

Reflection—After the action is executed (or rejected by the human veterinarian), observe the resulting changes in disease indicators (e.g., new PCR positives, behavioral deterioration). Compute a reward signal (e.g., timeliness of detection, cost savings, reduced transmission). Update the agent's internal policy or value function – this is how the agent learns and self-evolves.

This closed-loop workflow is illustrated in Figure 3.

**Figure 3:**
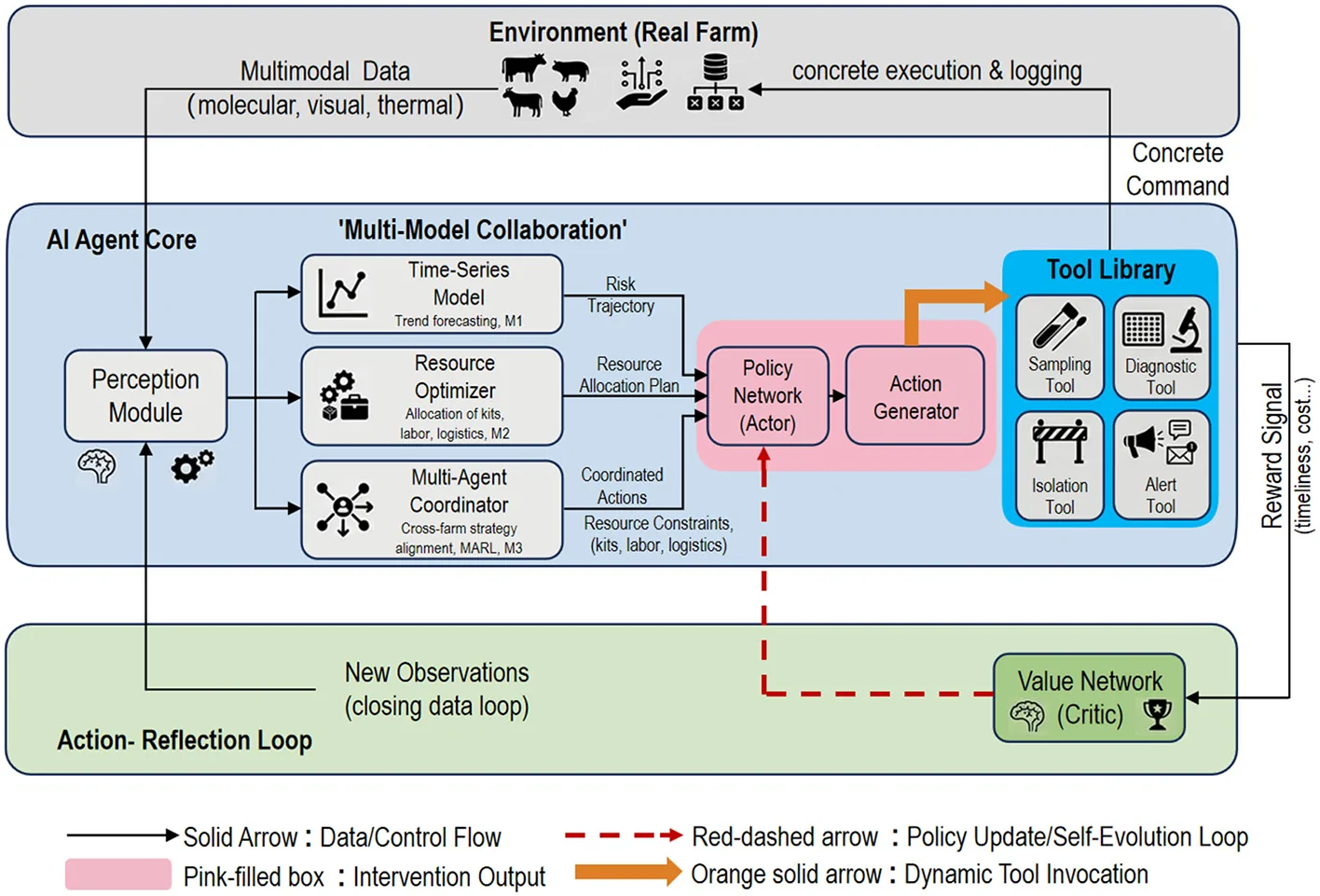
Architectural framework of the AI agent system for animal epidemic monitoring and intervention. The framework consists of three main domains: the real farm environment **(top)**, the AI agent core **(middle)**, and the action–reflection loop **(bottom)**. Within the AI agent core, the perception module ingests multimodal data (molecular, visual, thermal) and feeds a structured state into a multi-model collaboration layer comprising three specialized models: a time-series model for epidemic trend forecasting (M1), a resource optimizer for allocation of kits, labor, and logistics (M2), and a multi-agent coordinator for cross-farm strategy alignment using MARL (M3). Their outputs are integrated by the policy network (actor), which generates concrete intervention commands. The agent dynamically invokes tools from the tool library (sampling, diagnostic, alert) to execute the intervention (pink box). After execution, the environment returns new observations (closing the data loop) and a reward signal (e.g., timeliness, cost reduction) that is evaluated by the value network (critic). The critic updates the policy network via the red dashed arrow, forming the reflection/self-evolution loop. Solid arrows represent data or control flow; orange arrows denote dynamic tool invocation. This architecture highlights two key differentiators of AI agents from traditional open-loop predictors: multi-model collaboration and dynamic tool use.

#### 4.1.1 Why AI agents, not just traditional AI?

Traditional AI methods (including supervised learning for fusion analysis) operate as open-loop predictors: they map inputs to outputs (e.g., risk probability) and stop. They do not propose actions, respect dynamic resource constraints, or learn from the consequences of decisions. Table 2 contrasts the two paradigms in the context of an African swine fever (ASF) scenario.

**Table 2:** The two paradigms in the context of an African swine fever (ASF) scenario.

Aspect	Traditional AI (e.g., LSTM risk classifier)	AI agent (this review)
Output	“Risk score = 0.3”	“Take swab from pig #247, allocate 1 RT-PCR kit, expect result in 4 h.”
Handling of constraints	Not considered	Explicitly modeled (kits, labor, logistics)
Action feedback	None – model is static	Yes – reward signal updates policy
Self-evolution	Requires retraining on new data	Online learning / policy gradient updates
Role in outbreak	Decision support for humans	Autonomous recommendation + adaptive re-planning

Thus, the key reason to deploy AI agents instead of traditional AI is not merely better data fusion – it is the ability to close the loop from perception to action under realistic veterinary constraints, and to continuously improve from the outcomes of previous interventions.

### 4.2 Learning epidemic dynamics using time series models

Within the agent's Reasoning module, time-series models serve as the core engine for translating raw data streams into forward-looking risk estimates. However, unlike conventional forecasting models that produce a single prediction, the agent embeds these predictions into a policy network that decides which action to take next. Unlike traditional static detection methods that only reflect the health status at a single point, time series methods can capture the dynamic evolution of epidemics, which is crucial for responding to rapidly changing veterinary field conditions (61).

Short-term and medium-term incidence trends are particularly important for veterinary epidemiology (62), where epidemic data are often sparse, heterogeneous, or delayed in reporting. For instance, in the monitoring of Porcine Reproductive and Respiratory Syndrome (PRRS), sequence data and laboratory test records typically exhibit high variability; in wildlife disease monitoring (such as for African antelopes), direct sampling is logistically difficult and costly, leading to incomplete data (63). Such data limitations severely reduce the performance of traditional statistical models, making accurate epidemic prediction difficult.

Time series models combined with self-supervised pre-training frameworks can effectively address the data challenges in the veterinary field. For instance, Hidden Markov Models (HMM) have been successfully applied to infer the disease progression status of dairy cows from fecal sampling data, and can even convert unstructured fecal shedding data into actionable risk assessment information, even in cases where the disease is latent and has a long incubation period (64). The combination of Long Short-Term Memory (LSTM) models and Bayesian anomaly detection methods has demonstrated superior performance in identifying PRRS epidemics using sequence data, outperforming traditional regression and control chart methods (65). Various time series models such as ARIMA and LSTM have been employed to predict the trend of monkeypox prevalence, effectively mining the patterns of epidemic changes in global and country-specific monkeypox case data, and demonstrating stable and outstanding predictive performance in datasets from regions with different epidemic characteristics (66). The integrated MaxEnt-SARIMA coupled ecological time series model has demonstrated outstanding performance in regional early warning of zoonotic brucellosis. It can capture the transmission patterns of the disease by integrating livestock density, environmental factors, and epidemiological data, providing more accurate early warning basis for disease risk control in livestock breeding scenarios than traditional models (67).

In agent-based monitoring systems, these time series models serve as core learning modules, transforming continuous monitoring data streams into forward-looking risk assessments. By embedding time series predictions into the agent architecture, prediction is no longer an isolated analysis stage but a continuous adaptive process that evolves with changes in the farming environment and epidemic dynamics (68). For example, an intelligent early warning system integrating time series knowledge graphs and Internet of Things (IoT) devices can continuously collect real-time data (such as animal body temperature, activity levels, and environmental parameters) and update risk assessments in real time, providing more adaptive early warnings for livestock epidemics (69). Importantly, these time-series outputs are not the final product; they are fed directly into the agent's decision module (Section 4.3), where they are combined with resource availability and intervention costs to select an action.

### 4.3 Decision-oriented agents under resource constraints

Based on the dynamic learning of epidemic situations through time series models and reinforcement learning algorithms, precise prediction of the epidemic transmission trajectory has become a fundamental function of animal disease agents. However, in actual veterinary epidemic prevention work, effective epidemic intelligence not only requires accurate prediction but also feasible intervention decisions under real resource constraints. Monitoring and control are constrained by multiple factors, including sampling capacity, laboratory processing capacity, personnel allocation, and economic costs. Simulation studies on African swine fever have shown that even with available diagnostic tools, improper allocation of sampling and testing resources can lead to significant delays in epidemic detection, thereby severely weakening the effectiveness of early detection (30).

Decision-oriented agents explicitly incorporate these constraints into the reasoning process. Such models not only predict the epidemic trend but also evaluate different monitoring and intervention strategies, balancing epidemic risk and operational feasibility. For instance, the agent may recommend prioritizing the testing of specific high-risk pig herds or choosing other sample types to alleviate the pressure on laboratories. In the context of animal health management systems, epidemic prevention and control often require coordination across different farms, regions, and even administrative boundaries. Multi-agent reinforcement learning (MARL) provides a powerful mathematical framework for such distributed decision-making. By representing each jurisdiction or production unit as an autonomous agent, the MARL system can capture cross-jurisdictional epidemiological interactions while optimizing the allocation of limited resources (such as diagnostic kits or vaccines) under strict global budget constraints (70).

Furthermore, resource allocation in epidemic management is inherently a multi-objective optimization problem. Interventions that solely aim to maximize disease control effects often come with unsustainable logistical or economic burdens. Recent studies have shown that multi-agent reinforcement learning can achieve Pareto optimality among competing objectives, such as balancing the pressure on the medical system and reducing socioeconomic impacts. Translating this to the veterinary epidemiology context, such dual-objective reward functions enable agents to dynamically balance culling intensity, control of animal movement, and the economic sustainability of the livestock industry, effectively avoiding suboptimal strategies resulting from ignoring real operational conditions.

Moreover, both pathogen transmission and resource availability exhibit significant spatial heterogeneity, which requires dynamic adjustment capabilities in resource allocation strategies. Traditional static allocation models often struggle to adapt to the dynamic changes of the epidemic over time. Multi-agent reinforcement learning algorithms model spatial resource allocation as a cooperative game among regional agents, enabling them to make real-time collaborative decisions based on continuous observation of local infection status and dynamically deploy limited control resources to emerging epidemic hotspots (71).

Overall, a crucial differentiator of AI agents from classical operations research or supervised learning is their ability to perform sequential actions with reflection. In a typical farm outbreak scenario, the agent does not produce a single static plan. Instead, it follows an iterative loop:

At time *t*, the agent perceives state *S*_*t*_ (infection probabilities, remaining PCR kits, veterinarian availability).

It executes action *A*_*t*_ (e.g., “sample 5 high-risk animals”).

After the sampling results return at time t+Δt, the agent receives a reward *R*_*t*+1_ (e.g., +1 if a true positive was detected earlier than expected, −0.1 if resources were wasted on negative animals).

The agent updates its policy using reinforcement learning (e.g., PPO, DQN) and transitions to state *S*_*t*+1_.

This process is self-evolving: if the agent observes that its recommended sampling strategy consistently misses positives because of an unexpected clinical presentation, it will automatically adjust its internal belief distribution and shift sampling priorities in subsequent steps. No retraining of a separate static model is required. Such online adaptation is impossible for traditional AI pipelines that lack an action-feedback interface.

Thus, regional resource allocation is no longer a static bottleneck but becomes a dynamic control variable in the prediction-optimization feedback loop. This decision-oriented perspective is crucial for transforming AI prediction results into practical epidemic prevention outcomes - if logistical and resource constraints are ignored, even highly accurate predictions will be difficult to translate into improved animal disease outcomes.

## 5 Discussion and future perspectives

### 5.1 Intelligent-agent-enabled epidemic management in veterinary medicine

The evidence reviewed here supports an emerging technical foundation for intelligent-agent-enabled animal epidemic management, but the maturity of individual components remains uneven. Molecular diagnostics, biosensors, imaging, thermal monitoring, behavior recognition, and time-series forecasting have each been demonstrated in animal-health or livestock settings, whereas fully integrated systems that autonomously connect perception, resource-aware action, outcome evaluation, and online policy adaptation remain largely prospective. We therefore distinguish between technologies already validated for specific sensing or prediction tasks and the closed-loop agent architecture proposed in this review. The latter should be viewed as a research framework whose farm-level utility, safety, robustness, and cost-effectiveness still require prospective validation.

Beyond terrestrial livestock systems, recent reviews have also described the growing use of artificial intelligence in aquaculture for fish disease diagnosis, predictive modeling, and management decision support (72–74). This evidence from aquatic animal health broadens the application context of AI-enabled animal health management and suggests that similar data-driven principles may be adapted across diverse animal production systems. At the same time, these aquaculture studies emphasize challenges including fragmented datasets, interpretability, field adoption, and One Health integration, reinforcing the need for careful validation before more autonomous closed-loop systems are considered for routine use.

### 5.2 Future research directions

Building on these component-level advances, Future research should first focus on the upgrade of the input end. Existing inputs still have problems such as discrete sampling, insufficient image resolution, and inconsistent cross-platform standards. Subsequently, more sensitive pathogen detection, higher-frequency non-invasive sampling, clearer and more interference-resistant visual recognition systems can be developed, and unified data labeling, collection frequency, and quality control standards can be established. For example, high-definition RGB cameras can be used to identify small skin lesions, thermal imaging can be used to capture early inflammation, depth cameras can be used to quantify body condition and posture changes, and multi-omics and pathogen load data can improve the ability of early risk stratification. The key point is to enable the agent to obtain earlier, more accurate, and more continuous state inputs, rather than relying solely on single detection results.

Secondly, future research should focus on solving the problems of time, resources, and multi-modal collaboration. Animal disease prevention and control is not a simple prediction task but rather a matter of achieving the shortest response time under limited sampling personnel, reagent kits, laboratory throughput, and logistics. Therefore, the agent needs to incorporate time series prediction and resource constraints into decision-making: when to sample, which animals to sample, which type of detection to prioritize, and whether to initiate isolation or mobile laboratories should all be dynamically generated by the model based on risk and cost. The core of multi-modal collaboration is not simply stacking data but automatically adjusting weights in different scenarios: when images are occluded, increase the weight of behavior and temperature data; when laboratory throughput is insufficient, prioritize high-risk individuals or non-invasive group samples.

Finally, future research should improve the decision-making system and negative feedback regulation mechanism. Currently, many studies still remain at the risk scoring stage, and agents that can continuously correct strategies based on intervention results are still in the early stages. Subsequently, reinforcement learning and human-machine collaboration mechanisms can be introduced, with detection positive rate, early detection time, resource consumption, false alarms and missed detections, animal welfare, and economic losses as positive and negative feedback signals. Positive feedback can reward strategies that detect infections earlier and control transmission at lower costs; negative feedback is used to punish ineffective sampling, excessive isolation, resource waste, or missed high-risk individuals. If validated prospectively under field conditions, such feedback regulation could allow future agents to evolve from one-time prediction tools toward more adaptive and resource-aware epidemic management systems.

## 6 Conclusion

In conclusion, current evidence supports many of the sensing, diagnostic, phenotypic, and predictive components required for AI-enabled veterinary disease surveillance, but fully integrated closed-loop AI agents remain largely prospective. Future progress will depend not only on improvements in individual algorithms, but also on reliable multi-scale inputs, multimodal temporal integration, resource-aware decision support, and validated feedback mechanisms. Prospective field studies will be essential to determine the safety, robustness, cost-effectiveness, and practical value of these systems before their routine implementation in veterinary epidemic management.

## References

[1] Coelho IMP Paiva MT da Costa AJA Nicolino RR. African swine fever: spread and seasonal patterns worldwide. Prev Vet Med. (2025) 235:106401. doi: 10.1016/j.prevetmed.2024.106401 39700862

[2] Hayman DTS Adisasmito WB Almuhairi S Behravesh CB Bilivogui P Bukachi SA . Developing One Health surveillance systems. One Health. (2023) 17:100617. doi: 10.1016/j.onehlt.2023.100617 PMC10665171 38024258

[3] Sharan M Vijay D Yadav JP Bedi JS Dhaka P. Surveillance and response strategies for zoonotic diseases: a comprehensive review. Sci One Health. (2023) 2:100050. doi: 10.1016/j.soh.2023.100050 PMC11262259 39077041

[4] Li Z Li D Dong J Zhu Q Zuo Y Pu J . Integrated health surveillance and early warning systems in China under the one health perspective: progress and challenges. Sci One Health. (2025) 4:100132. doi: 10.1016/j.soh.2025.100132 PMC12766100 41498121

[5] Mediouni S Ndione C Parmley EJ Poder TG Carabin H Aenishaenslin C. Systematic review on evaluation tools applicable to One Health surveillance systems: a call for adapted methodology. One Health. (2025) 20:100995. doi: 10.1016/j.onehlt.2025.100995 PMC11893302 40071275

[6] Khoshnood S Pakzad R Koupaei M Shirani M Araghi A Irani GM . Prevalence, diagnosis, and manifestations of brucellosis: a systematic review and meta-analysis. Front Vet Sci. (2022) 9:976215. doi: 10.3389/fvets.2022.976215 PMC9813401 36619963

[7] Schillings J Bennett R Rose DC. Exploring the potential of precision livestock farming technologies to help address farm animal welfare. Front Anim Sci. (2021) 2:639678. doi: 10.3389/fanim.2021.639678

[8] Bao J Xie QJ. Artificial intelligence in animal farming: a systematic literature review. J Clean Prod. (2022) 331:129956. doi: 10.1016/j.jclepro.2021.129956

[9] Shapiro JT Cazeau G DiBiagio R Dupuy C Morignat E Dorea F . Current practice and future directions in syndromic surveillance for animal health: a scoping review and analysis. Prev Vet Med. (2025) 241:106532. doi: 10.1016/j.prevetmed.2025.106532 40327964

[10] Neethirajan S Kemp B. Digital phenotyping in livestock farming. Animals. (2021) 11:2009. doi: 10.3390/ani11072009 PMC8300347 34359137

[11] Gómez Y Stygar AH Boumans IJMM Bokkers EAM Pedersen LJ Niemi JK . A systematic review on validated precision livestock farming technologies for pig production and its potential to assess animal welfare. Front Vet Sci. (2021) 8: 660565. doi: 10.3389/fvets.2021.660565 PMC8160240 34055949

[12] Belák S Thoren P LeBlanc N Viljoen G. Advances in viral disease diagnostic and molecular epidemiological technologies. Expert Rev Mol Diagn. (2009) 9:367–81. doi: 10.1586/erm.09.19 PMC7105750 19435457

[13] Wozniakowski G Truszczynski M Pejsak Z. Increasing importance of genomics in recognition of infectious animal diseases taking into account DIE requirements. Med Weter. (2017) 73:10–4. doi: 10.21521/mw.5630

[14] Bold D Souza-Neto JA Gombo-Ochir D Gaudreault NN Meekins DA McDowell CD . Rapid identification of ASFV, CSFV and FMDV from Mongolian outbreaks with MinION short amplicon sequencing. Pathogens. (2023) 12:533. doi: 10.3390/pathogens12040533 PMC10140976 37111419

[15] Cong X Tong F Liu H Zhu Y Tan N Gu F . Establishment of a novel double-antibody sandwich fluorescence microsphere immunochromatographic test strip for rapid detection of swine acute diarrhea syndrome coronavirus (SADS-CoV) infection. Front Cell Infect Microbiol. (2025) 15:1461845. doi: 10.3389/fcimb.2025.1461845 PMC11906713 40093537

[16] Zhao WH Qin H Li W Zhang Z Bing Q Wang S . Development of a dual-channel QDM immunosensor for rapid and highly sensitive quantitative detection of antibodies against African swine fever and pseudorabies virus. Microchem J. (2025) 214:113969. doi: 10.1016/j.microc.2025.113969

[17] Ren Q Wang Y Ma H Xie J Jin J Tian R . Recent advances in lateral flow immunoassay for rapid diagnosis of viral diseases. Transbound Emerg Dis. (2026) 2026:5701806. doi: 10.1155/tbed/5701806 PMC12790179 41522489

[18] Shome R Patra S Sahib MM Shanmugam G Skariah S Shamshad S . Chimeric protein A/G conjugate based lateral flow assay for the rapid detection of brucellosis in multiple animal species. J Immunol Methods. (2025) 539:113845. doi: 10.1016/j.jim.2025.113845 40049236

[19] Zhao Y Zhang L Wang A Zhou D. Biosensor technology: advances and applications in livestock infectious disease diagnosis. Vet Sci. (2025) 12:23. doi: 10.3390/vetsci12010023 PMC11769105 39852898

[20] Vidic J Manzano M Chang CM Jaffrezic-Renault N. Advanced biosensors for detection of pathogens related to livestock and poultry. Vet Res. (2017) 48:11. doi: 10.1186/s13567-017-0418-5 PMC5320782 28222780

[21] Gattani A Mandal S Agrawal A Patel P Jain AK Singh P . CRISPR-based electrochemical biosensors for animal health: recent advances. Prog Biophys Mol Biol. (2024) 193:7–18. doi: 10.1016/j.pbiomolbio.2024.09.001 39237013

[22] Perez AA Vazquez-Meves G Hunter ME. Early detection of wildlife disease pathogens using CRISPR-Cas system methods. CRISPR J. (2024) 7:327–42. doi: 10.1089/crispr.2024.0030 39479796

[23] Notsu K Hala ED Mitoma S Wu X Norimine J Sekiguchi S. Correction for Notsu et al., “Identifying pathogen and allele type simultaneously in a single well using droplet digital PCR.” *mSphere*. (2023) 8:e0020823. doi: 10.1128/msphere.00208-23 PMC10470587 37310719

[24] Eman S Din RMU Zafar MH Zhang M Wen X Ma J . Technologies in biomarker discovery for animal diseases: mechanisms, classification, and diagnostic applications. Animals. (2025) 15:3132. doi: 10.3390/ani15213132 PMC12609278 41227463

[25] Eshawu AB Gunda TJ Kumar B Matta S. Advancements in sample preparation and extraction techniques for metabolomic analysis of animal fluids. Microchem J. (2025) 218:115259. doi: 10.1016/j.microc.2025.115259

[26] Jadav MM. Karmbir, Joshi V. Saliva as alternative diagnostic biofluid for non-invasive health and disease appraisal in domestic and wild animals: a review of salivary biomarkers and sialochemistry. Acta Vet Hung. (2025) 73:264–76. doi: 10.1556/004.2025.01192 41186609

[27] Schupbach BS Davis MS Jennings TD Dixon AL Renter DG Nickell JS. Comparison of a novel bovine respiratory disease prediction technology and an automated animal disease detection technology to traditional methods in a U.S. feedlot. Transl Anim Sci. (2025) 9:txaf067. doi: 10.1093/tas/txaf067 PMC12161071 40511290

[28] Hobbs EC Colling A Gurung RB Allen J. The potential of diagnostic point-of-care tests (POCTs) for infectious and zoonotic animal diseases in developing countries: technical, regulatory and sociocultural considerations. Transbound Emerg Dis. (2021) 68:1835–49. doi: 10.1111/tbed.13880 PMC8359337 33058533

[29] Ochwo S Perez AM Perez Aguirreburualde MS. Beyond accuracy: leveraging ASSURED criteria for field evaluation of point-of-care tests for food animal diseases. Front Vet Sci. (2023) 10:1239111. doi: 10.3389/fvets.2023.1239111 PMC10500061 37720479

[30] Galvis JA Satici MY Sykes AL O'Hara KC Rochette L Roberts D . Estimating sampling and laboratory capacity for a simulated African swine fever outbreak in the United States. Prev Vet Med. (2025) 239:106529. doi: 10.1016/j.prevetmed.2025.106529 40203482

[31] Arulmozhi E Bhujel A Moon BE Kim HT. The application of cameras in precision pig farming: an overview for swine-keeping professionals. Animals. (2021) 11:2343. doi: 10.3390/ani11082343 PMC8388688 34438800

[32] Rao R Qadri S Kashif R. Realtime monitoring of animal behavior using deep learning models. J Agric Environ Int Dev. (2025) 119:125–48. doi: 10.36253/jaeid-16397

[33] Luo J Li F Luo X Zhu X. 13th International Conference on Information Science and Technology (ICIST). Cairo: IEEE (2023). p. 311–6. doi: 10.1109/ICIST59754.2023.10367190

[34] Hao W Xu S-A Shu H Li H Han M Li F . VM-RTDETR: advancing DETR with vision state-space duality and multi-scale fusion for robust pig detection. Animals. (2025) 15:3328. doi: 10.3390/ani15223328 PMC12649245 41302036

[35] Shirke A Saifuddin A Luthra A Li J Williams T Hu X . Tracking Grow-Finish Pigs Across Large Pens Using Multiple Cameras. arXiv:2111.10971. (2021). doi: 10.48550/arXiv.2111.10971

[36] Satoła A Bauer EA. Predicting subclinical ketosis in dairy cows using machine learning techniques. Animals. (2021) 11:2131. doi: 10.3390/ani11072131 PMC8300340 34359259

[37] Marchegiani S Chiappini S Choudhury MAM Guangxin E Trombetta MF Pasquini M . Affordable 3D technologies for contactless cattle morphometry: a comparative pilot trial of smartphone-based LiDAR, photogrammetry and neural surface reconstruction models. Agriculture. (2025) 15:2567. doi: 10.3390/agriculture15242567

[38] Shi W Dai B Shen W Sun Y Zhao K Zhang Y. Automatic estimation of dairy cow body condition score based on attention-guided 3D point cloud feature extraction. Comput Electron Agric. (2023) 206:107666. doi: 10.1016/j.compag.2023.107666

[39] Taechachokevivat N Kou B Zhang T Montes ME Boerman JP Doucette JS . Evaluating the performance of herd-specific long short-term memory models to identify automated health alerts associated with a ketosis diagnosis in early-lactation cows. J Dairy Sci. (2024) 107:11489–501. doi: 10.3168/jds.2023-24513 39245172

[40] Sabathier R Mitra NJ Novotny D. Computer Vision – ECCV 2024. Vol. 15137. Switzerland: Springer Nature (2025). p. 270–87. doi: 10.1007/978-3-031-72986-7_16

[41] Asay BC Edwards BB Andrews J Ramey ME Richard J Poodell BK . Digital image analysis of heterogeneous tuberculosis pulmonary pathology in non-clinical animal models using deep convolutional neural networks. Sci Rep. (2020) 10:6047. doi: 10.1038/s41598-020-62960-6 PMC7142129 32269234

[42] Tavolara TE Niazi MKK Ginese M Piedra-Mora C Gatti DM Beamer G . Automatic discovery of clinically interpretable imaging biomarkers for Mycobacterium tuberculosis supersusceptibility using deep learning. EBioMedicine. (2020) 62:103094. doi: 10.1016/j.ebiom.2020.103094 PMC7658666 33166789

[43] Kim E Fischetti AJ Sreetharan P Weltman JG Fox PR. Comparison of artificial intelligence to the veterinary radiologist's diagnosis of canine cardiogenic pulmonary edema. Vet Radiol Ultrasound. (2022) 63:292–7. doi: 10.1111/vru.13062 35048445

[44] Muhammad Saqib S Iqbal M Othman MTB Shahazad T Ghadi YY Al-Amro S . Lumpy skin disease diagnosis in cattle: a deep learning approach optimized with RMSProp and MobileNetV2. PLoS ONE. (2024) 19:e0302862. doi: 10.1371/journal.pone.0302862 PMC11299804 39102387

[45] Mukadam S Patil H. Skin cancer classification framework using enhanced super resolution generative adversarial network and custom convolutional neural network. Appl Sci. (2023) 13:1210. doi: 10.3390/app13021210

[46] Kim T Visbal-Onufrak MA Konger RL Kim YL. Data-driven imaging of tissue inflammation using RGB-based hyperspectral reconstruction toward personal monitoring of dermatologic health. Biomed Opt Express. (2017) 8:5282–96. doi: 10.1364/BOE.8.005282 PMC5695970 29188120

[47] Reza MN Lee K-H Habineza E Samsuzzaman Kyoung H Choi YK . RGB-based machine vision for enhanced pig disease symptoms monitoring and health management: a review. J Anim Sci Technol. (2025) 67:17–42. doi: 10.5187/jast.2024.e111 PMC11833201 39974778

[48] Sharvanthika KS Ramya K Vanitha CN. Hybrid deep learning approach for early mastitis detection in dairy cattle using convolutional neural networks and vision transformers. In: 2024 International Conference on Emerging Research in Computational Science (ICERCS). Coimbatore: IEEE (2024). p. 1–8. doi: 10.1109/ICERCS63125.2024.10895431

[49] Mozafari M Law AJ Goubran RA Green JR. Respiratory rate estimation from thermal video data using spatio-temporal deep learning. Sensors. (2024) 24:6386. doi: 10.3390/s24196386 PMC11479072 39409425

[50] Fodor I van der Sluis M Jacobs M de Klerk B Bouwman AC Ellen ED. Automated pose estimation reveals walking characteristics associated with lameness in broilers. Poult Sci. (2023) 102:102787. doi: 10.1016/j.psj.2023.102787 PMC10404698 37302328

[51] Eckhardt R Arablouei R Ingham A McCosker K Bernhardt H. Livestock behaviour forecasting via generative artificial intelligence. Smart Agric Technol. (2025) 11:100987. doi: 10.1016/j.atech.2025.100987

[52] Gendron G Chen Y Rogers M Liu Y Azhar M Heidari S . Behaviour modelling of social animals via causal structure discovery and graph neural networks. In: Proceedings of the 23rd international conference on autonomous agents and multiagent systems (AAMAS 2024). Auckland, New Zealand (2024). p. 2276–8.

[53] Lencioni GC Sousa RVd Sardinha EJdS Corrêa RR Zanella AJ. Pain assessment in horses using automatic facial expression recognition through deep learning-based modelling. PLoS ONE. (2021) 16:e0258672. doi: 10.1371/journal.pone.0258672 PMC8525760 34665834

[54] Kobayashi K Sakamoto N Miyazaki Y Yamamoto M Murata T. Automated pain assessment based on facial expression of free-moving mice. PNAS Nexus. (2025) 4:pgaf352. doi: 10.1093/pnasnexus/pgaf352 PMC12617409 41245089

[55] Han X Wang Y-T Feng J-L Deng C Chen Z-H Huang Y-A . A survey of transformer-based multimodal pre-trained models. Neurocomputing. (2023) 515:89–106. doi: 10.1016/j.neucom.2022.09.136

[56] Ahmed MR Haque R Rahman SMA Reza AW Siddique N Wang H. Vision-audio multimodal object recognition using hybrid and tensor fusion techniques. Inf Fusion. (2026) 126:103667. doi: 10.1016/j.inffus.2025.103667

[57] Lee S Ahn Y Shin JW. Multimodal emotion recognition using modality-wise knowledge distillation. Sensors. (2025) 25:6341. doi: 10.3390/s25206341 PMC12567929 41157397

[58] Burti S Banzato T CoghlanS Wodzinski M Bendazzoli M Zotti A. Artificial intelligence in veterinary diagnostic imaging: perspectives and limitations. Res Vet Sci. (2024) 175:105317. doi: 10.1016/j.rvsc.2024.105317 38843690

[59] Dusza P Banzato T Burti S Bendazzoli M Muller H Wodzinski M. Comparative evaluation of CAM methods for enhancing explainability in veterinary radiography. Sci Rep. (2025) 15:29690. doi: 10.1038/s41598-025-14060-6 PMC12350829 40804451

[60] Marić K Gvozdanović K Kušec ID Kušec G Margeta V. Smart pig farms: integration and application of digital technologies in pig production. Agriculture. (2025) 15:937. doi: 10.3390/agriculture15090937

[61] Lamanna M Bovo M Cavallini D. Wearable collar technologies for dairy cows: a systematized review of the current applications and future innovations in precision livestock farming. Animals. (2025) 15:458. doi: 10.3390/ani15030458 PMC11815998 39943229

[62] Wang Y Huang G Chen C Li Q Xu X. A comparative study of time series foundation models for hand, foot, and mouth disease forecasting: TimesFM, Moirai, and traditional approaches. Front Public Health. (2025) 13:1634138. doi: 10.3389/fpubh.2025.1634138 PMC12507834 41080839

[63] Barroso P López-Olvera JR Kiluba wa Kiluba T Gortázar C. Overcoming the limitations of wildlife disease monitoring. Res Dir One Health. (2024) 2:1–37. doi: 10.1017/one.2023.16

[64] Ceres KM Schukken YH Grohn YT. Characterizing infectious disease progression through discrete states using hidden Markov models. PLoS ONE. (2020) 15:e0242683. doi: 10.1371/journal.pone.0242683 PMC7678993 33216809

[65] Diaz-Cao JM Liu X Kim J Clavijo MJ Martinez-Lopez B. Evaluation of the application of sequence data to the identification of outbreaks of disease using anomaly detection methods. Vet Res. (2023) 54:75. doi: 10.1186/s13567-023-01197-3 PMC10492347 37684632

[66] Wei W Wang G Tao X Luo Q Chen L Bao X . Time series prediction for the epidemic trends of monkeypox using the ARIMA, exponential smoothing, GM (1, 1) and LSTM deep learning methods. J Gen Virol. (2023) 104:001839. doi: 10.1099/jgv.0.001839 37022959

[67] Chen Q Abudunaibi B Zhao X Bahati J Wang Y Rui J . Spatiotemporal early warning of human brucellosis in Hubei Province using coupled ecological-time series models. Zoonoses Public Health. (2026) 73:244–61. doi: 10.1111/zph.70046 PMC13053619 41741367

[68] Chhabra A Singh SK Sharma A Kumar S Gupta BB Arya V . Sustainable and intelligent time-series models for epidemic disease forecasting and analysis. Sustain Technol Entrep. (2024) 3:100064. doi: 10.1016/j.stae.2023.100064

[69] Bhandwalkar K Jadhav D. Artificial intelligence and internet of things enabled systems for smart veterinary disease detection. Discov Artif Intell. (2026) 6:418. doi: 10.1007/s44163-026-01173-8

[70] Sharma D Shah A Gopalappa C. A multi-agent reinforcement learning framework for public health decision analysis. Healthc Anal. (2025) 8:100436. doi: 10.1016/j.health.2025.100436 PMC12858148 41624295

[71] Zong K Luo C. Reinforcement learning based framework for COVID-19 resource allocation. Comput Ind Eng. (2022) 167:107960. doi: 10.1016/j.cie.2022.107960 PMC8800507 35125625

[72] Öz M Ustuner E Cifci S Budak F Ileri E Dikel S. Artificial intelligence for fish disease diagnosis and management: innovations, challenges, and one health implications. Aquac Int. (2025) 33:652. doi: 10.1007/s10499-025-02352-7

[73] Fernandes S Dmello A. Artificial intelligence in the aquaculture industry: current state, challenges and future directions. Aquaculture. (2025) 598:742048. doi: 10.1016/j.aquaculture.2024.742048

[74] Öz M Üstüner E. Artificial intelligence for blue transformation: a review of predictive modeling and decision support systems in sustainable aquaculture. Sustain Dev. (2026). doi: 10.1002/sd.71290

